# Exploring Knowledge and Perceptions of Menopause Among Peri-Menopausal Women in Greece: Insights into the Role of Mobile Applications in Menopausal Management

**DOI:** 10.7759/cureus.86397

**Published:** 2025-06-19

**Authors:** Evgenia-Ioanna Papadima, Tonia Vassilakou, Themos Grigoriadis, Theodoros Fouskas, Sofia Ivanidou, Stella Roidi, Lina Michala

**Affiliations:** 1 1st Department of Obstetrics and Gynecology, National and Kapodistrian University of Athens School of Medicine, Athens, GRC; 2 Department of Public Health Policy, School of Public Health, University of West Attica, Athens, GRC

**Keywords:** climacteric, menopause, menopause mobile application, perimenopause, qualitative study design

## Abstract

Menopause marks a natural phase in a woman's life and affects a significant portion of Greek women aged 40-69 and beyond. With increasing digitization, mobile health apps are transforming healthcare delivery, offering accessible and cost-effective support. This study explored the experiences of Greek peri-menopausal and menopausal women through focus groups, aiming to inform the development of a culturally appropriate mobile application. Participants, recruited via snowball sampling, completed questionnaires and engaged in online discussions moderated by researchers. Transcripts were analyzed using Braun and Clarke’s thematic framework. Key findings revealed widespread gaps in menopause knowledge, especially regarding its stages and symptoms. Women reported experiencing hot flushes, weight gain, mood disturbances, and vaginal dryness, often uncertain if symptoms were menopause-related. Menopausal hormone therapy was met with reluctance and misconceptions. Menopause was often perceived as a “taboo” subject, rarely discussed even with doctors, and likened to puberty in terms of emotional and social impact. Women reported workplace challenges, including stress, burnout, and ageism. While none had used menopause-specific apps, many were familiar with health-related apps and expressed interest in a menopause app offering nutritional, psychological, and lifestyle guidance. Most preferred Greek-language content, and views were mixed on whether the app should be free. Overall, the study underscores the need for educational, supportive, and culturally sensitive digital tools. A mobile app could bridge information gaps and offer a private, empowering space for Greek women navigating menopause.

## Introduction

Menopause is a natural stage in the aging process, which occurs 12 months after a woman's last menstrual period, marking the conclusion of her reproductive years [1]. With increasing life expectancy, women now spend a substantial proportion of their lives in menopause [2]. According to the Hellenic Statistical Authority, women make up 51.1% of the Greek population, with approximately 42.6% aged between 40 and 69, indicating that a significant proportion are experiencing perimenopause, menopause, or post-menopause [3].

The North American Menopause Society (NAMS) in their Recommendations for Clinical Care of Midlife Women states that menopause can be viewed as a sentinel event that affords a unique opportunity for a dialogue between women and their healthcare providers to evaluate and improve health-related practices (Level II) [4].

Digital health is an innovative and challenging proposition. Due to global trends in digitization, modern societies increasingly rely on digital tools, and this also changes people’s attitudes and expectations towards the provided medical care, which now tends to be digitalized [5]. Mobile health (mHealth) is a subset of electronic health (eHealth) and is defined as the provision of health services with the support of mobile devices, such as mobile phones or Personal Digital Assistants [6]. Many mobile applications have been developed, offering a variety of features and capabilities, such as self-monitoring or recording, turning mobile devices into diagnostic tools [6].

Contemporary peri-menopausal Greek women are likely to utilize smartphones and have access to the internet. Notably, there were 8.9 million internet users in Greece in January 2024 [7]. Information and communication technologies (ICTs) and digital services, such as smartphones, offer a powerful means of educating patients by enhancing behavior change.

The number of medical or health-related apps is growing rapidly in the major app stores (Apple App Store and Google Play Store). The use of smartphones changes the delivery of patient education in healthcare through convenient and personalized interventions [8]. In addition, smartphone applications have lower costs, reduce patient burden, and overcome some limitations of traditional face-to-face interventions [8-10].

A search conducted in application stores utilizing the Greek term for menopause, ‘εμμηνοπαυση’ (assessed on August 7, 2024), revealed a lack of relevant applications pertaining to menopause in the Greek language. Furthermore, a research study conducted in 2019 identified various English-language mHealth applications that address menopausal issues, concluding that the involvement of healthcare providers is essential in the development of these applications. This underscores the need for culturally and linguistically appropriate resources to support women navigating menopause, as well as the importance of collaborative development efforts in mHealth [11]. According to the same study, the first application specifically designed for the menopause was introduced a decade ago [11].

The purpose of this qualitative study was to investigate common concerns among women approaching or navigating menopause, so as to create an easy-to-use mobile application relevant to Greek peri-menopausal and menopausal women. As a secondary outcome, we aimed at collecting their views on the relevance and utility of such an application. 

The focus group method is a type of group interview involving several participants and a moderator. It highlights a specific topic and fosters interaction among participants to co-create meaning [12]. This method blends aspects of both group interviews, which cover various topics, and focused interviews that target individuals with specific experiences related to a situation. While focused interviews can be conducted individually or in groups, the focus group method uniquely emphasizes group interaction, offering a more concentrated format than standard group interviews and facilitating deeper discussions among participants.

## Materials and methods

Women were recruited for the study through a menopause clinic and a primary health care center. A snowball sampling technique was employed to facilitate the recruitment of additional participants. This method allowed for the identification and inclusion of women who may have otherwise been overlooked, thereby enhancing the study's representativeness and depth. 

Informed consent was obtained from all participants, who were made aware that the focus group discussions would be recorded. Prior to their participation, each individual completed a comprehensive 26-item questionnaire designed to gather pertinent demographic information, details regarding menstrual history, and insights into their knowledge and utilization of menopause-related mobile applications. Additionally, the questionnaire included inquiries about recent visits to an obstetrician and gynecologist, or a family practitioner. The questionnaire was electronically transcribed using Google Forms® and disseminated online prior to the meetings (refer to Table 1). 

**Table 1:** Introductory online questionnaire Values are count and percent Ob/Gyn: Obstetrics/Gynaecology; GP: General Practitioner The data were collected from 9 to 28 February 2024.

Are you still having periods (last menstrual period within past year)	
Yes	15 (55.6%)
No	12 (44.4%)
When was your last period?	
1 month ago	12 (80.0%)
3 months ago	3 (20.0%)
How would you describe your most recent menstrual period?	
Heavy/more blood than usual	3 (20.0%)
Less blood than usual	2 (13.3%)
Lasted more than usual	1 (6.7%)
Lasted less than usual	4 (26.7%)
Stable cycle	5 (33.3%)
Do you use any mobile phone applications to track your periods?	
Yes	5 (33.3%)
No	10 (66.7%)
Do you use any applications for menopause management?	
Yes	1 (3.7%)
No	26 (96.3%)
Did you visit an Ob/Gyn over the previous year?	
Yes	19 (70.4%)
No	8 (29.6)
Did you discuss menopausal issues with an Ob/Gyn?	
Yes	17 (63.0%)
No	10 (37.0%)
Do you have a family doctor?	
Yes	16 (59.3%)
No	11 (40.7%)
Have you discussed menopausal issues with your family doctor?	
Yes	10 (37.0%)
No	17 (63.0%)

A comprehensive semi-structured interview guide was developed by Evgenia-Ioanna Papadima (EIP) and Lina Michala (LM), drawing upon pertinent literature and findings from prior research conducted by our group [13,14]. Additionally, LM and EIP served as moderators and facilitators, as well as interviewers, during the three focus group sessions. The first and second focus group was formed by 10 participants each, whereas the third comprised of seven women.

Tentative dates were proposed for both weekdays and weekends in order to enhance participation rates. The focus groups were conducted online via the Zoom® platform, and the recordings were transcribed into text using an online word processing application. The focus groups were held on February 9, 18, and 28, 2024. A fourth focus group was not conducted, as a preliminary assessment indicated that theoretical data saturation had been achieved.

The documents were pseudonymized and subsequently uploaded to the Taguette® platform [15], enabling the highlighting, tagging, and thematic categorization of salient messages expressed by participants. To enhance the presentation of results, each participant was assigned random two-letter initials. 

Thematic analysis was conducted following the framework established by Braun and Clarke [16] by researcher EIP, under the supervision of LM and TF. Initially, both researchers engaged in an immersive review of the data to develop a comprehensive understanding of the material. Subsequently, they generated initial codes by identifying salient features within the dataset. These codes were then systematically categorized into overarching themes, which were critically evaluated for their relevance to the research questions posed. The refinement process involved multiple iterations, resulting in well-defined themes accompanied by precise definitions and nomenclature. Finally, the findings of the analysis were articulated through a narrative, supported by relevant quotations, ensuring robust interpretations of the data.

The representative quotations were translated into English by EIP and LM. Subsequently, these translations were evaluated by SR, who had access to the original statements and the complete transcript. SR ensured that the original meaning and conceptual integrity of the quotations were preserved throughout the translation process.

The research study obtained ethical clearance from the Ethics Committee of the National and Kapodistrian University of Athens Medical School, as documented under protocol number 374/5.11.20.

## Results

In this study, a total of 27 Greek women, aged between 47 and 55 years, participated as subjects. The general socio-demographic characteristics of the sample are delineated in Table 2.

**Table 2:** General characteristics of the sample ^a^ Values are mean and standard deviation of mean ^b^Values are count and percent BMI: Body Mass Index The data were collected from 9 to 28 February 2024.

Demographics and general characteristics (n=27)	^a^Mean±SD or ^b^Count (percentage)
Age^a^(years)	50.5 ± 2.65
Weight^a^ (Kg)	69.5 ± 13.96
Height^a^ (cm)	1.64 ± 0.06
BMI^a^ (Kg/m^2^)	25.9 ± 4.79
ΒΜΙcategories ^b^	
Underweight	1 (3.8%)
Normal weight	11 (42.3%)
Overweight or Obese	14 (53.8%)
Smoking ^b^	
Yes	9 (33.3%)
No	18 (66.7%)
Children ^b^	
0	3 (11.1%)
1	9 (33.3%)
2-3	14 (51.8%)
4 or more	1 (3.7%)
Marital status ^b^	
Unmarried	3 (11.1%)
Married	19 (70.4%)
Divorced	5 (18.5%)
Education level ^b^	
Secondary education (High school)	4 (14.8%)
Tertiary education (College/ University)	7 (25.9%)
Postgraduate degree (Master /PhD)	16 (59.3%)
Occupation level ^b^	
In employment (public or private employee, freelance)	23 (85.2%)
Not-working (unemployed/ housewife/ retired)	4 (14.8%)
Residence ^b^	
Athens and region of Attica	21 (77.8%)
Rural	4 (14.8%)
Living temporarily abroad	2 (7.4%)

The thematic analysis yielded nine distinct thematic sections, each contributing to a comprehensive understanding of the subject matter (Table 3).

**Table 3:** Thematic analysis

Theme	Subthemes
Sources of information on menopause	Family, healthcare professionals, online sources; limited and superficial information; difficulty discussing menopause with peers.
Menopausal stage	Confusion around definitions; lack of awareness about screening tests and preventative care; predominant focus on symptoms.
Symptoms	Vasomotor symptoms, weight gain, mood swings, libido issues, vaginal dryness, sleep disorders, memory loss, and positive changes like reduced migraines.
Menopausal hormone therapy (MHT) and other remedies	Lack of support or encouragement from doctors; reluctance to use MHT; use of herbal remedies and multivitamins; interest in complementary therapies.
Effect on family/social circle	Menopause perceived as a natural transition; impacts family dynamics; lack of understanding from social circle; desire for empathy and awareness.
Effect on work	Workplace stress, burnout, age-related discrimination; calls for menopause-related workplace rights similar to pregnancy.
Knowledge and use of mobile applications	Low awareness of menopause apps; interest in symptom tracking and information; concerns about data privacy and tech adoption.
Contents of application	Desired features: nutrition and exercise guidance, symptom tracking, lab value explanations, chat or support function, interactive features, and personalization.
Language and price of use	Preference for Greek language; mixed opinions on payment vs. ad-supported models; support for flexible access and trial options.

Sources of information on menopause

The primary sources of information identified were family members, predominantly mothers, as well as healthcare professionals, specifically Obstetricians and Gynaecologists (Ob/Gyn) and General Practitioners (GPs). GL 52 years old mentioned:

‘…I was discussing, it with my mother, who has gone through (the change) relatively young. I remember how she was, so (…) I was asking my mother more and so little by little I realized what was going to happen and that I will have to face it coolly...’ 

Women within the second focus group reported that reading articles mostly online was very helpful for them. However, participants agreed that the information that they received was limited. EL 49 years old noted:

‘…on the sites, I read the same things, which are quite trivial. For example, it tells you that you have certain symptoms, like dryness, weight gain around the abdomen, and so on and so forth... but the questions I have, which go a bit further, are not answered by these sites.’ 

A general consensus in all focus groups was that women were not well informed and wanted to have access to more specific menopausal issues. ME 51 years old highlighted: 

‘During the climacteric period and menopause, you are much more in the dark, I mean I would like to have more information and clearly an app could have that role. …First of all, there are many symptoms, I don’t know whether they derive from this (meaning the climacteric and menopause) and you may be going through them and not know why you’re still going through them.’ 

A common observation among one third of the participants is that, although they pose questions, they do so without experiencing anxiety; they do not exhibit 'anxious asking'. Furthermore, in all focus groups at least two participants would indicate that menopause is a subject they struggle to discuss openly with their friends or peers. This notion was generally accepted by most women. PH 49 years old said: 

‘I don’t now… You don’t go out in groups, let’s say, you don’t talk at any given moment for the climacteric and menopause, right?’

However, one woman disagreed and mentioned that now women in radio and television admit more overtly that they are menopausal. Furthermore, she reported also having discussions within her peer group. IR 49 years old said:

‘……then so many, friends also enter this period a little sooner or later, so you either learn something new or you overanalyze what you have already seen in yourself.’ 

Menopausal stage

There was a general ignorance on the definitions of climacteric and menopause, so most women could not identify their menopausal stage. During the second focus group, a participant genuinely wanted to know whether she was menopausal and only two participants of that group agreed they really knew. KA 49 years old gave her point of view: 

‘I think, what I know is that…- in non-medical terms I mean-, I know it…purely empirically or from the general knowledge that exists, (the menopause) is the…the point from where one clearly begins to see changes in one’s cycle. And the various other changes that one can combine with other hormonal let’s say symptoms, that one can see in herself.’ 

A difficult question, where women remained mostly silent and required extensive prompting was: ‘What are the things that you are afraid of during this phase? What makes you anxious about this phase? What are the things that you don’t know and you would like to know?’. Women wanted to know more regarding preventative measures during menopause. Moreover, they were not aware of the specific screening tests that they should do (i.e. bone density measurement, smear tests). A woman expressed a desire to gain a deeper understanding of hormonal replacement therapy, while another sought information regarding weight management. However, the predominant focus of discussion among the group revolved around the various symptoms they were experiencing.

Symptoms

Women had a strong urge to talk about their symptoms. The discussion in the first focus group in particular was monopolized by this subject. Predominantly, they had night sweats and hot flushes. Women didn’t really know how to deal with them and only one reported some tips, like layering or using a fan, a tip which was found useful by other members of the focus group. Speaking with their own words FA 55 years old mentioned: 

‘…these hot flushes are incredible! The flare-ups I feel especially in the summer! I am sitting and I am sweating!’ 

However, there are certain symptoms associated with menopause that can be challenging for women to recognize and attribute to this transition, one of which is weight gain. Many women report noticing alterations in their body weight, with some acknowledging a shift in their overall body shape. SM 49 years old, was wondering about weight changes: 

‘For example, weight gain, right? You gain weight… and we have all faced it, you gain very easily, you lose hard… How long does this thing last? From now on will we always be like this? So should we be counting carbohydrates by the gram? Or will the body somehow return to how it was?’ 

Another common concern were mood disorders. DA 47 years old shared: 

‘…what I found out is that I have several ups and downs in my mood, big ups and big downs…I mean, it comes in waves of mood and generally ranges from days, where I don’t have, let’s say,… I am not willing to do anything. Everything eventually goes on of course! I’m just not in the mood myself. And then, there are other days when I am at my best again!’

One to two participants in each focus group mentioned a decrease in libido and vaginal dryness. ZO 48 years old shares her own concern: 

‘What I noticed besides the vaginal dryness that I had and...I had, I had a lot,...I didn’t feel like having sex at all and it was very intense, and this is still going on now.’ 

Women were wondering if there was a hereditary pattern in their symptoms. In fact, EL 49 years old asked: 

‘Can I ask one more question (…) Whether and to what extent does menopause have any elements of heredity, i.e. do I have the same symptoms that my mom did for example?’

Some other symptoms and conditions they reported were bloating, sleep disorders, memory loss, and menstrual irregularities. Finally, women agreed on certain positive aspect of menopause on conditions such as migraines and anemia, which have a tendency to improve in the post-menopausal period.

Menopausal hormone therapy (MHT) and other remedies

A woman believed that MHT is primarily used for the alleviation of severe, rather than mild symptoms. Another asked if it could work as a preventative treatment. Women wanted to know if they could take hormonal replacement, why it is prescribed and when is the ideal time to start taking it. For example, SM 49 years old said: 

‘….in Greece, I think there is ‘a big taboo’ regarding hormones. My gynecologist had suggested it to me. I didn’t take it because I had already gone through the peak of the symptoms (she describes it as a 'whole mountain'), I didn’t have symptoms anymore, I thought that the bone density tests are good and so on, and I didn’t think it made any sense, but I still think there is a taboo in Greece regarding hormone treatment..’ 

Most women expressed that they did not receive positive reinforcement from their doctors regarding hormone replacement therapy. There was a general reluctance and bias, either from the women themselves or from their doctors, to prescribe MHT. The risks associated with MHT were often overemphasized, leading some women, like SM, to decline treatment despite having an absolute indication for it due to a diagnosis of early menopause. Notably, none of the women were on hormone treatment, nor were they using vaginal estrogens. However, they did admit to using lubricants during intercourse. 

Another topic was their awareness of herbal remedies and complementary health approaches to treat menopause symptoms. Some women were taking multivitamins to treat menopause symptoms, either prescribed from their doctor or at their own initiative and some had used herbal remedies in the past. In fact, VA 54 years old, described: 

‘The doctor had recommended some herbal remedies, herbal medicines, how should I say it? Which might have helped me, but I don’t know what it would have been like if I hadn’t…if I hadn’t taken them. What would be the difference - how would it be? You can’t know exactly what would have happened to you, to say that it was successful. I think that they had been helpful.’

Meditation, acupuncture, yoga and herbal remedies such as agnus cactus were mentioned by participants as complementary health approaches and women were mostly willing to use them, but some were worried there might be interactions with other prescribed medication. For example MI 54 years old mentioned:

‘I am afraid to use alternative herbal remedies, because I am now taking thyroxine……I am afraid to use another medication. I haven’t been told by a doctor if I can, right?’ 

Effect on family/social circle

The primary emphasis of this thematic section centers on the perceptions and attitudes held by others regarding menopause and menopausal women. This exploration seeks to understand the societal narratives and cultural constructs that shape the experiences of women in this transitional phase of life, as well as the implications these perceptions have for women's health and well-being. A recurring sentiment was that menopause is a natural transition, like puberty, rather than a curse or disease. This parallel was brought forward in the first and third focus group and was found very appropriate by the remaining of the participants. Using their own words, HEE 49 year old said:

‘Menopause is not a kind of illness, it is like my child’s puberty, I don’t just treat it with paracetamol. It’s like other phases we go through. I think it is important for reconciliation with the part of us that is evolving and maturing or requires a ‘so to speak’ … ‘treatment’ in inverted commas. … We may have symptoms, but we are not patients.’ 

There was a consensus during all focus groups that menopause affects not only the woman in question, but also their social environment, which is often unable to deal with the issues relating to menopause. More specifically, three women believed that menopause affected their family and children and they wished for them to be more informed, to be more sympathetic and supportive. Six women felt that their environment was sorry for them on occasions and treated them like being 'on their period'. In fact, RO 54 years old mentioned: 

‘Even my children said: ’We can’t talk to you, you are so on edge ’. But how can you explain to children what’s going on in your inner world? (Laughs)… That’s why I said that there has to be information, because you must be accepted with your symptoms in your social environment.’ 

Again MI 54 y agreed: 

‘...But there are too many people around you again, that… don’t understand you; how you are feeling’ 

Finally, GA 55 y concluded: 

‘Menopause is very, very similar to puberty! Very much so! I’m like...and it’s not just us women who experience it right? Men sometimes have this type of symptoms as well. Nevertheless, it is a period that resembles adolescence, and I say it clearly. Just as in adolescence you reorganize your existence, likewise in the climacteric it is as if you are reorganizing who you are…In other words it also coincides with a change in our family structure. I don’t know if this applies to everyone, but certainly for several of us. So you redefine your existence.’ 

Effect on work

Four participants faced significant stress in the workplace, often expressing a desire for more supportive or accommodating treatment. They reported experiencing burn out, with some recounting even instances of bulling. Listening to their opinions was extremely enlightening. At first, KA 49 years old said: 

‘…So, the question arises now in the workplace, to what extent menopause, like pregnancy, can serve as a reason…..for a woman to be granted certain leaves from work, or to be recognized, essentially, as a reason, like pregnancy or childbirth, legally defined as a valid legal reason for corresponding rights, similar to those that exist during a woman's reproductive years’.

Continuing, GL 52 y reported

‘…So, even in the workplace, because unfortunately we spend most of our time there, they try to undermine us and make us feel small. I personally experience this a lot in my workplace because I am the oldest, (…) and I constantly face….. bullying about my age....’ 

Finally, GA, 55 years old highlighted: 

‘ …the women in the British National Health care System, who work, … at 45-50 years of age are resigning, almost collectively, precisely because they have suffered burn out due to menopause and their work…’

Knowledge and use of mobile applications

Participants in our study were not particularly aware of specific applications dedicated to the menopause, despite reporting good experience with smartphone applications relating to other gynecological conditions. Two participants had used calendar applications to record their period or menstruation-related symptoms. Three mentioned having visited online sites or using other applications for previous life stages, such as pregnancy. The only woman (ME, 49 y) who had used a menopause-related application shared her point of view: 

‘I am still in pre-climacteric or climacteric, if I am saying this right. I have most of the symptoms that you have discussed… I use an application that is available on my smart watch. And it has helped me in terms of recording, let’s say, when is the (menstrual) cycle, when it happens, eh its duration, the basic things.’

Seven women found that a mobile application for menopause would be useful in the future for many reasons. IO 50y mentioned

‘Yes. I think it would be useful if there was something to save a lot of phone calls with a doctor, so as not to bother them with various questions, that may not be purely medical but could also be psychological.’ 

However, two women expressed skepticism about recording information in an application. Indeed PH 50y said:

‘And in general I’m a bit(…) I don’t know if I’ll say opposed(…) I’m a bit cautious with apps like this and social media, meaning I am not too interested.’

Contents of application

Half of the participants expressed a wish for the mobile application to include nutritional information and exercise instructions. They wanted to have access to psychology information, special support and tips. They also were keen to receive information regarding herbal remedies and alternative medicines that would relieve them from symptoms. IO 50 years old mentioned: 

‘So yes, I find such an application useful, to include nutritional information to psychological or other consequences an early menopause or menopause in general can have.’

In continuation EV 47 years old agreed: 

‘… I would definitely be interested in an….application that will guide us in some way. i.e. having a variety of information, either in terms of general symptoms or treatment. Because …. I don’t think there is enough information…it’s not a topic where we can easily find the information we need, so definitely an application would be useful in general…’ 

Regarding having information on nutrition, EL 49 years old suggested:

‘Nutrition is important, because one thinks they know, but essentially, they don’t have proper guidance, that 'these are good to include in one’s diet'. So it would be important to have something on nutrition. I think it wouldn’t replace a dedicated nutrition app- it would just include some basic features, like dietary advice. And of course there should be a correlation with calories, as that’s also important.’ 

EL also felt that it would be useful to have information on normal values for basic laboratory tests, such as full blood counts. So as to see how they get affected by menopause. The presence of concerning indicators suggesting the existence of abnormal values: ‘And perhaps, I don’t know how interesting it would be, but you could include some basic medical indicators, i.e. white blood cells, red blood cells, saying that usually women in the menopause are usually at this level; Let’s say their range usually falls around here. So, it would be interesting to include this diagrammatically with a curve(…)it’s very important to have something like this, so that someone can see the bigger picture.’ 

Moreover, six women expressed a wish to chat with other women through the application, which they would ideally want to be interactive. RO 54 years old proposed: 

‘in addition to information let’s go to communication. Don’t forget that many women are alone. They experience loneliness, so it might be a way for them to learn, to absorb information and to be able to talk to someone else.’

Two of the participants also emphasized the importance of having an interactive platform in diverse topics. They highlighted the need for using evidence-based sources, pre-prepared Q&A materials, and a dedicated help desk. Additionally, they stressed the value of having reputable staff available to address inquiries, connect users with experts, and offer tailored advice for a more personalized experience. HE 49 years old explains:

‘Personally, I find it useful, like all applications, if it covers a range of topics and does not focus solely on medical information; it should address the various aspects we’ve discussed. What is very important to me is the credibility of the content. The information should come from reputable sources. Additionally, it would be beneficial if there were pre-prepared Q&As, addressing questions that have genuinely concerned people so that I can access them easily. The third point is that I wouldn't propose a chat room with other women because anyone could stray off-topic there. I would prefer a help desk, where someone could provide advice in a reasonable timeframe (…) but supported by a team that can answer questions. What many people may be missing is personalization, rather than generalization’.

One woman requested audio-visual material and found that the actual discussion taking place during the focus group was very useful and enjoyable. All women in this particular focus group were in agreement with this statement. MA 47 years proposed: 

‘…I was thinking, while we were talking, that what we do is also very interesting today, so maybe an application could have something audio. That is experiences from women and how they experienced the climacteric, the doctor-specialist to talk to you…that is, not only to read…., Because, there is a power in storytelling, in this topic-through more personal stories, that someone would want to share, or through something that a doctor might prefer to express in their own words rather than in a diagram or a Q&A format, and so on.’ 

Finally, three women wanted to receive cosmetic advice for hair and skin along with antiaging tips. One woman asked for webinars and another for meditation tips. Another wanted the app to have the ability to grade the symptoms like traffic lights (red, green, yellow). Another participant focused on anonymity of interaction through the application. Women asked for links to specialists, but they thought it would be unprofessional to receive specific medical advice through the app. Also, they wanted to have some kind of guarantee that their data will be protected. 

Language and price of use

A significant preference among participants emerged for Greek as the language of communication in the context of the application. This inclination suggests a potential cultural or pragmatic alignment with the language, warranting further exploration of the factors influencing this choice.

ME, 51 years old: 

‘I personally don’t have any problem if it is in Greek or in English. However, I believe that when it is addressed to…it is made for Greece, it should be in Greek!’ 

There was a difference of opinion regarding if women should pay to have the mobile application. Participants did not favor the fact that a free application would likely be sponsored by advertisements. To explore this further, a prompting question was used: What would you prefer? A free application supported by advertisements, or one available for a small fee, such as €0.99, but completely ad-free? In response, seven women favored the latter option. Several practical solutions were proposed during the discussions. For example, to offer two versions of the application. An ad-supported version and an ad-free version for a small fee. Another idea was to introduce a free trial period, allowing users to experience the app before deciding on a purchase. AL 45 years old, proposed: 

‘One idea would be to provide a free trial period, 15 days, a month, so that the user can see if it is really something that…she will use… …’

SO, 52 years old, added: 

I also think it’s better for the application to be free. After all, we already have so many expenses. I believe that just because something is presented to us in an advertisement doesn’t mean we have to follow it. Instead, through discussions about the products that interest us-perhaps with a gynecologist or similar professionals-we can make informed purchase decisions. I find it more flexible and quicker not to deal with cards and other payment processes. It’s not about the €0.99 fee itself but about the hassle involved, especially when time is limited. You forget, you leave it. So, I prefer to have it freely accessible whenever I need it and decide later if an advertisement influences me enough to act on it.'

## Discussion

This study, to our knowledge, is the first qualitative research project on menopause conducted among Greek women, utilizing focus groups as the primary methodological approach. The aim of the research was to uncover and understand concerns and experiences of menopausal women, shedding light on the possible challenges they face. By exploring the perspectives of participants, the study provides insight into the social, emotional, and informational gaps that influence their journey through menopause, offering valuable information that can be used in future interventions and support mechanisms. From our part, these insights will be used for the development of a mobile application, enriched with relevant and up-to-date information to address the needs of menopausal women effectively.

Focus groups are an excellent method for examining the gap between health knowledge and health behaviors [12,16,17]. Our experience proved that it also provided a supportive and safe environment, where women felt comfortable discussing their concerns and seeking knowledge. The qualitative approach was well suited to exploring complex, subjective experiences that are often underrepresented in quantitative research.

Just over one-third of the participants were menopausal, having experienced a menstrual period more than a year previously, while the remaining women were in the climacteric phase. In each focus group, there were a few participants who primarily listened and rarely contributed to the discussion. While they typically agreed with the opinions of others, they were hesitant to share their own perspectives. It is interesting to note that these quieter participants were more often in the climacteric stage rather than fully menopausal. Plausible explanations for their reluctance to engage might be that they did not yet perceive themselves as significantly affected by the issues being discussed, or that they had limited experience with menopausal symptoms, leading to their hesitation in speaking. 

However, the majority of women were motivated and willing to discuss their concerns and symptoms, providing us with a depth of information regarding their perceptions. For example, they demonstrated a limited understanding of menopause and often felt confused and overwhelmed by the physical and emotional changes they were experiencing. Also, they often did not know the exact stage of the climacteric or menopause they were in. However, the transitional changes of menopause were repeatedly paralleled to the changes of adolescence. During the meetings, we stressed that menopause is a ‘process and not an event’ [18]. According to its definition, we consider a woman as menopausal 12 months after her last menstrual period, which also marks the end of the menstrual cycles [1]. Peri-menopause begins around the age of 45 and ends one year after menopause. Finally, post-menopause refers to the final part of this transitional period, which starts from menopause and reaches beyond age 65 [18]. 

The most commonly reported symptoms in this study were hot flushes, reduced libido, and weight gain. This finding aligns with previous research, which has highlighted hot flushes, vaginal dryness, fatigue, and insomnia as prevalent symptoms [19]. Menopause in general [20] and the severity of symptoms affect quality of life negatively [21]. But there may be more to just an effect on quality of life: According to the “Women’s Health Initiative,” having ≥2 moderate or severe menopausal symptoms was associated with a higher risk for cardiovascular disease, recommending that they are used as a marker for this [22]. Moreover, women in our study repeatedly complained of weight gain; therefore, getting lifestyle advice for better cardiovascular health is important, and we plan to integrate this information in a dedicated section of the application. 

Indeed, women had a strong desire for nutritional and exercise advice, although many were unaware that regular physical activity could also help manage menopausal symptoms. Furthermore, they requested comprehensive information on managing hair loss and weight gain, as well as details about MHT and alternative health approaches, like yoga and meditation.

Regarding MHT, there was a general reluctance and overly increased fear of side effects. Women felt that, since menopause is a stage that everyone goes through, it is better to avoid using medication. This is an important issue to address. Despite risks in older women, MHT should be offered to women entering the menopause early, and there is a clear indication for its use for the treatment of vasomotor symptoms [1]. 

Despite reluctance to receive MHT, a recurring theme was the anxiety associated with aging. Yet, many women were actively working toward acceptance and reconciliation with change. In fact, women were in agreement that the menopause signified an opportunity for redefining oneself. And on a positive note, the discussions revealed unexpected benefits of the menopause, including improvements in anemia and migraines. 

None of the participants reported having a family doctor with whom they discussed menopausal concerns. This indicates a potential gap in primary healthcare engagement on menopause-related issues, emphasizing the need for better integration of family doctors into women’s health management during this transition. Importantly, these findings do not diminish the significance of the original research question; quite the opposite-they add depth to it. 

Notably, many women in our study viewed menopause as a topic they felt unable to openly discuss with others. This contrasts with findings from a study of middle-aged Korean women, who perceived menopause as a form of illness [19] and often sought information from friends, family members, or the internet [19]. Such cultural differences underscore the diverse ways menopause is understood and experienced globally and highlight the importance of creating dedicated means for particular cultural and ethnic groups.

Many women experience menopause while still being employed, and their symptoms may have an impact on their work efficiency. The Health and Employment After Fifty study looked at a cohort precisely in this setting and concluded that employment did not affect the prevalence of women’s symptoms and that employers should be made aware of the particularities of this group of women [23]. This is a conclusion that often came up in our study, as we identified a common statement among women, having expectations for a better working environment, more understanding and acceptance, and commitment to investing in their work potential. Our findings echo evidence that menopause is shaped by lifelong social and economic disadvantage. For women in casual work, support must extend beyond workplace policies to address broader structural inequalities [24].

For our team, one of the most insightful findings was the participants’ complete lack of awareness regarding the existence of menopause-related mobile applications. Not a single woman in the group had previously encountered or used such a tool. Nevertheless, some were familiar with health-related applications, acknowledging, for example, the value of tracking their periods, currently or in the past. This practice is encouraged in the perimenopause by NAMS [4].

Women were open to the idea of diverse content formats in an application, including both written and audio material, whether narrated by other women sharing their experiences or by healthcare specialists offering expert guidance. A noteworthy example of a relevant resource is Healthtalk. org, which provides qualitative, user-centered material on a variety of health topics, including the menopause [25]. 

Participants expressed mixed opinions regarding their willingness to pay for a mobile application. While they hesitated at the idea of a paid service, they acknowledged using other paid mobile applications. Opinions converged however on the importance of offering the application in Greek, ensuring accessibility and ease of use. All participants showed enthusiasm for the app's potential benefits and would be willing to engage with it and provide feedback once it becomes available.

Study limitations

This study has limitations that should be considered when interpreting the findings. The use of focus groups may have led to underrepresentation of more reserved participants, particularly those in the climacteric stage who did not yet strongly identify with menopausal concerns. Additionally, the relatively small sample size restricts the breadth of perspectives captured and may not adequately reflect the full range of menopausal experiences. Finally, the sample, composed solely of Greek women, limits the generalizability of results to other populations. 

## Conclusions

Menopause remains a culturally sensitive and often "taboo" subject, frequently likened to puberty in terms of its transformative impact on family and professional dynamics. Despite its significance, women often hesitate to seek information or support, and the resources available are perceived as insufficient. This study, along with previous research from our team has provided with the necessary background to introduce a smartphone application for Greek peri-menopausal women. 

## Appendices

**Table 4:** Topic guide: Semi-structured cognitive interview guide

Interview Sections	Specific Questions
Introduction Greetings/Welcome	‘Hello! Thank you for joining us today to talk about peri-menopause. My name is E.I.P, I am a family doctor and my thesis is about creating a mobile application for climacteric and menopause, therefore we are having this focus group today, along with my supervisor L.M.’ ‘Let me inform you that during the interview I’ll be taking notes and that LM will record our conversation. Do you agree?’ ‘I would also like your permission to speak in the singular, do you agree?’ ‘The working group will last about an hour. Let’s get started, shall we?
Main Part Knowledge Questions: Questions related to behaviors/experiences, questions regarding opinions or attitudes, and probing questions	‘What do you know about climacteric/ menopause’? ‘What would you like to know more about climacteric / menopause?’ ‘What do you think your gaps are?’ ‘We have asked you in google form questionnaire if you know any mobile application for menopause and if you have ever tried one? ‘What is the most difficult for you in relation to menopause?’ ‘What scares you/ what worries you the most about menopause?’ ‘Have you discussed with your gynaecologist for climacteric issues or symptoms, for example menstrual period disturbances or vaginal dryness?’ ‘Where do you get information about menopause?’ ‘What do you consider to be the most reliable sources of information on menopause issues?’ ‘What do you think for mobile applications for menopause?’ ‘Do you think mobile applications for menopause could be useful? If yes, why?’ ‘Do you think that women would try to use a mobile application for menopause? Are you willing to try one / our application in the future?’ ‘What would you like to provide a special menopausal mobile application? What information do you need the app to have?’ ‘Do you think it is important the application to be in Greek language?’ ‘Do you think that it is important this mobile application to be available for free, with no payment?’ ‘What do you mean? Can you tell me more about…?’ ‘Participants in previous groups wanted to learn more about the symptoms and how to deal with them, for example menstrual period disturbances, night’s sweats, hot flushes, sleeping disorders, vaginal dryness or weight gain. What do you think?’ ‘Participants in previous focus groups considered important to be an application made from a reputable team, like from National and Kapodistrian University of Athens, Athens Medical School. What do you think?’ ‘Participants in previous focus groups considered important that the application will have ready material in Q&As. What did this sound like to you?’ ‘Participants in previous focus groups considered important that the application will not only provide medical information, but also will provide interaction, like chatting with other women, or with medical staff. What do you think?’ ‘Other things that the mobile application could provide , would be information for the menopausal stage you are, medical information for health issues like HRT, period calendar for climacteric women, symptoms calendar, weight control histogram, nutrition calendar, psychological advices, nutritional tips or exercise tips. What do you think?’ ‘Would you like the mobile application to have information for complementary health approaches, like mindfulness or herbs?’ ‘Would you like the mobile application to inform you for aesthetic issues during peri-menopause, like facial botox or vaginal laser?’ ‘Would you like the application to use audio-visual material? To present other women’s experiences? Like i.e. healthtalk.org’? ‘If the application had advertises, what would you think? More specifically what type of ads would you prefer to have?’
Relaxation questions related to perceptions and feelings	‘How did you feel when the interview started?’ ‘Now that it is over how are you feeling?’
Closing goodbye	‘Thank you very much for joining this focus group for our study today and we wish you the best!’

**Table 5:** 26-item questionnaire

Question
I have read and understood the information about the study.
I understand that the session will be recorded. I give my permission for this.
I consent to the storage of transcribed information. I understand confidentiality will be maintained.
I understand that participation is voluntary and I may withdraw at any time.
I agree to take part in this focus group session.
Did you have a period in the past year?
When was your last period?
How would you describe your period (e.g., your last three cycles)?
Have you used or are you currently using a mobile app to track your period?
Do you know of any mobile applications for perimenopause or menopause?
If you know of a menopause app, do you use it?
Which menopause app do you use?
What do you track in the app?
Where did you learn about this menopause mobile app?
Have you visited your gynecologist in the past year?
Did you discuss menopause-related issues (e.g., menstrual disturbances, sleep disturbances, vaginal dryness)?
Are you registered with a general/family doctor?
Have you discussed menopause-related issues (e.g., osteoporosis) with him/her?
Date of birth
Place of residence
Education level
Employment status
Height (in meters)
Weight (in kilograms)
Number of children
Do you smoke?
